# Effect of noninvasive embryo viability testing versus conventional IVF on the live birth rate in IVF/ICSI patients: a study protocol for a double-blind, multicenter, randomized controlled trial

**DOI:** 10.1186/s12884-023-05892-z

**Published:** 2023-09-06

**Authors:** Yan-Fei Cheng, Cui-Lian Zhang, Yun Liu, Jian-Ping Ou, Lei Chen, Gui-Feng Cai, Zu Yang, Tian-Min Ye, Jun Wang, Juan-Ke Xie, Ping Xiong, Xi-Ya Zhang, Min Li, Wei-Biao Xu, Xiao-Qing Wang, Ling-Yin Kong, Bo Liang, Xiao-Hong Wang, Yue-Qiang Wang, Yuan-Qing Yao

**Affiliations:** 1 Shenzhen Key Laboratory of Fertility Regulation, Reproductive Medicine Center, The University of Hong Kong-Shenzhen Hospital, No. 1 Haiyuan Road, Shenzhen, 518053 Guangdong China; 2 Department of Obstetrics and Gynecology, Chinese PLA General Hospital, Beijing, 100853 China; 3 Reproductive Medical Center, Henan Provincial People’s Hospital, Zhengzhou, 450003 Henan China; 4 Center of Reproductive Medicine, 900th Hospital of the Joint Logistics Team, Fuzhou, 350009 Fujian China; 5 Reproductive Medical Center, The Third Affiliated Hospital of Sun Yat-Sen University, Guangzhou, 510630 Guangdong China; 6 Reproductive Medical Center, The Sixth Medical Center of Chinese PLA General Hospital, Beijing, 100048 China; 7 Reproductive Medical Center, Zhuhai Center for Maternal and Child Health Care, Zhuhai, 519001 Guangdong China; 8 Basecare Medical Device Co., Ltd, 218 Xinghu Street, Suzhou Industrial Park, Suzhou, 215000 Jiangsu China; 9 Reproductive Medical Center, Tangdu Hospital, Air Force Medical University, 569 Xinsi Rd., Baqiao District, Xi’an, 710038 Shaanxi China; 10 School of Life Sciences and Biotechnology, Shanghai Jiao Tong University, Shanghai, 200240 China; 11 School of Biology and Food Engineering, Changshu Institute of Technology, Changshu, 215506 Jiangsu China

**Keywords:** Noninvasive preimplantation genetic testing, Embryo, Live birth, Conventional IVF, Randomized controlled trial, Study protocol

## Abstract

**Background:**

Preimplantation genetic testing for aneuploidy (PGT-A) was demonstrated to be superior to conventional IVF in reducing the incidence of miscarriage and abnormal offspring after the first embryo transfer (ET). PGT-A requires several embryo trophectoderm cells, but its negative impacts on embryo development and long-term influence on the health conditions of conceived children have always been a concern. As an alternative, noninvasive PGT-A (niPGT-A) approaches using spent blastocyst culture medium (SBCM) achieved comparable accuracy with PGT-A in several pilot studies. The main objective of this study is to determine whether noninvasive embryo viability testing (niEVT) results in better clinical outcomes than conventional IVF after the first embryo transfer. Furthermore, we further investigated whether niEVT results in higher the live birth rate between women with advanced maternal age (AMA, > 35 years old) and young women or among patients for whom different fertilization protocols are adopted.

**Methods:**

This study will be a double-blind, multicenter, randomized controlled trial (RCT) studying patients of different ages (20–43 years) undergoing different fertilization protocols (in vitro fertilization [IVF] or intracytoplasmic sperm injection [ICSI]). We will enroll 1140 patients at eight reproductive medical centers over 24 months. Eligible patients should have at least two good-quality blastocysts (better than grade 4 CB). The primary outcome will be the live birth rate of the first embryo transfer (ET). Secondary outcomes will include the clinical pregnancy rate, ongoing pregnancy rate, miscarriage rate, cumulative live birth rate, ectopic pregnancy rate, and time to pregnancy.

**Discussion:**

In this study, patients who undergo noninvasive embryo viability testing (niEVT) will be compared to women treated by conventional IVF. We will determine the effects on the pregnancy rate, miscarriage rate, and live birth rate and adverse events. We will also investigate whether there is any difference in clinical outcomes among patients with different ages and fertilization protocols (IVF/ICSI). This trial will provide clinical evidence of the effect of noninvasive embryo viability testing on the clinical outcomes of the first embryo transfer.

**Trial registration:**

Chinese Clinical Trial Registry (ChiCTR) Identifier: ChiCTR2100051408. 9 September 2021.

## Background

Preimplantation genetic testing for aneuploidy (PGT-A) was demonstrated to lead to better clinical outcomes than conventional IVF (based on morphological assessment) in several clinical studies [1–3]. PGT-A can precisely detect embryonic chromosome abnormalities, effectively reduce pregnancy loss, and promote healthy live births in patients undergoing IVF cycles. For a long time, PGT-A was performed using samples taken from embryos in different developmental stages (cleavage stage, blastocyst stage) [4]. Trophectoderm biopsy (TE biopsy) is the most widely applied biopsy method compared to polar body and cleavage-stage biopsies in clinical practice. Some studies have claimed that TE biopsy does not affect embryo development potential [5, 6]. However, other works have reached the opposite conclusion [7, 8]. Despite contradictory opinions, trophectoderm biopsy (TE biopsy)-based PGT-A has been widely applied in IVF cycles worldwide. However, invasive biopsies may negatively affect embryo development, and their effect on the long-term health of offspring is unclear. In addition, embryo biopsy increases the financial burden on patients because it relies on professional equipment and skilled embryologists [9]. For these reasons, doctors and patients are eager for a noninvasive PGT-A (niPGT-A) protocol.

The identification of embryonic DNA in the blastocoel fluid (BF) and spent blastocyst culture medium (SBCM) provides an opportunity to determine the embryo chromosomal status in a noninvasive way [10, 11]. Because of advantages such as the existence of a larger volume of embryonic DNA, easier sample retrieval, and avoidance of any embryo manipulation, SBCM is more widely used in niPGT-A than BF. Since the publication of the first proof-of-concept study demonstrating the potential of niPGT-A in 2016 (Shamonki et al., 2016), several works have briefly compared the performance of niPGT-A (using SBCM) and PGT-A (using TE biopsy, the inner cell mass, and the whole blastocyst) [12–14]. These studies reached a general conclusion that niPGT-A has a comparable accuracy to PGT-A. Despite the obvious advantages of niPGT-A over PGT-A, the clinical benefit of niPGT-A needs to be further evaluated in strictly designed randomized controlled trials (RCTs) with large samples.

To our knowledge, there are three (two prospective studies and one retrospective study) studies reporting clinical assessments of niPGT-A in different patients [15–17]. Chen et al. developed a machine learning algorithm that was used for embryo grading based on niPGT-A results. The algorithm was validated to be effective in avoiding wastage of potentially competent embryos in a cohort of 266 patients (aged 20 ~ 45 years). Fang et al. reported the transfer of 52 embryos determined to be euploid by niPGT-A in 43 patients (aged 23 ~ 42 years) with or without chromosomal rearrangements. Xi et al. conducted a retrospective study that demonstrated that niPGT-A effectively improved the clinical outcomes in patients with both recurrent pregnancy loss (173 patients) and repeated implantation failure (100 patients) compared to conventional IVF. Although these studies provide preliminary insights into the clinical benefits of niPGT-A, they suffer from a series of limitations, including limited study centers (all three studies were conducted in a single clinical center), a lack of proper control, and relatively small sample sizes. Consequently, a double-blind, multicenter RCT with a large sample is required to achieve a solid conclusion regarding the clinical benefits of niPGT-A.

The aim of this study is to evaluate whether noninvasive embryo viability testing (niEVT) results in better clinical outcomes than conventional IVF. niEVT assesses embryo developmental potential by integrating niPGT-A results (priority standard) with morphological grading results (second standard). Conventional IVF predicts embryo developmental potential based only on morphological grading results. In addition, we will also investigate which subgroup of patients is more likely to benefit from niEVT by comparing the clinical outcomes of patients with different ages and fertilization protocols.

### Trial objectives

PGT-A was demonstrated to be a reliable method for detecting aneuploid embryos during IVF. Polar body, cleavage-stage, or trophectoderm biopsies are required to obtain samples from embryos. This invasive sampling process raises concerns regarding the possible negative impact on clinical outcomes. Noninvasive preimplantation genetic testing for aneuploidy (niPGT-A), which detects chromosomal abnormalities in the cell-free DNA (cfDNA) of spent blastocyst culture medium, is expected to be applied in clinical practice. However, it is not clear whether niPGT-A can achieve better clinical outcomes than conventional IVF. In this study, patients who undergo noninvasive embryo viability testing (niEVT) will be compared to women treated by conventional IVF. We will determine the effects on the pregnancy rate, miscarriage rate, and live birth rate and adverse events. We will also investigate whether there are any differences in the clinical outcomes among patients with different ages and fertilization protocols (IVF/ICSI).

## Methods

### Trial design

**Fig. 1:**
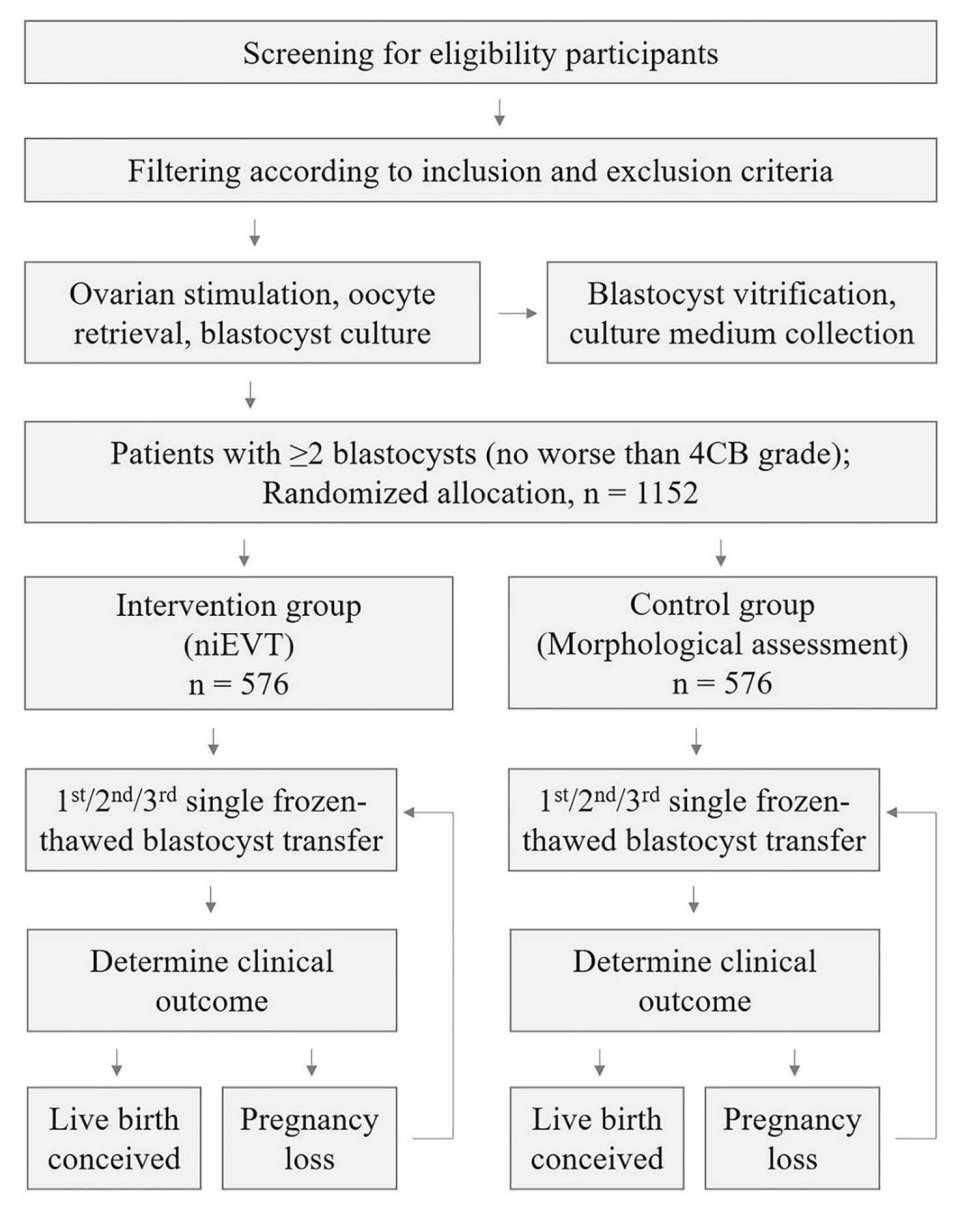
CONSORT flow diagram showing the procedure of this randomized controlled trial. Abbreviations: niEVT, noninvasive embryo viability testing; niPGT, noninvasive preimplantation genetic testing

This protocol is for a multicenter, double-blinded, randomized, controlled trial that will be conducted in China (Fig. 1). This protocol was revised several times by experts to form this final version before trial commencement. Patient recruiting is being carried out simultaneously in eight reproductive medical centers, including the First Medical Center of PLA General Hospital (PLAGH), the Sixth Medical Center of PLA General Hospital (6MC-PLAGH), the Air Force Medical University-Tangdu Hospital (Tangdu Hospital), the 900th Hospital of the Joint Logistics Team (900 Hospital), the Third Affiliated Hospital of Sun Yat-Sen University (3AH-SYSU), Henan Provence People’s Hospital (Henan-PPH), the University of Hong Kong-Shenzhen Hospital (HongKong-SH), and the Zhuhai Center for Maternal and Child Health Care (Zhuhai-CMCHC). Three rounds of clinical inspections will be conducted in every reproductive medical center by a trial management committee (TMC). The aim of the clinical inspections will be to ensure that the study is performed according to the protocol and that the data are collected and recorded in a timely manner.

### CONSORT statement

The reporting checklist for this protocol is based on the CONSORT guidelines [18].

### Trial status

The first patient was enrolled on December 1, 2021. The current version of the study protocol is version 2, dated April 12, 2021. The estimated study completion date is October 2023.

### Study participants

Eligible subjects must meet all the inclusion criteria. To be enrolled, the following criteria must be met: (1) Couples experiencing infertility who are undergoing their first IVF cycle; (2) Couples undergoing IVF or ICSI as the fertilization protocol; (3) Couples in which the female is aged 20–43 years; (4) Couples in which the female has a body mass index (BMI) of 18–25 kg/m^2^; (5) Couples who agree to the performance of single culture of all embryos on Day 4 (after fertilization); (6) Couples with ≥ 2 good-quality (morphological grade superior to 4 CB) blastocysts on Day 5/6 after fertilization; (7) Couples who agree to undergo single frozen-thawed blastocyst transfer; and (8) Couples in which both partners provide written informed consent.

Subjects who met any of the exclusion criteria will be excluded from the study. The exclusion criteria are as follows: (1) Couples (with any contraindications) who are unable to undergo in vitro fertilization; (2) Couples (with a known chromosomal abnormality) who are eligible for preimplantation genetic testing (PGT) cycles; (3) Couples in which the female has unexplained recurrent miscarriage (≥ 2 times); (4) Couples in which the female has diseases that affect the pregnancy outcome (e.g., untreated hydrosalpinx, untreated intrauterine infection, uterine fibroid diameter > 4 cm, pelvic and abdominal benign tumors > 4 cm, hCG daily endometrial thickness < 7 mm, pituitary tumors and malignant tumors of various tissues and organs); (5) Couples in which the female has a congenital uterine anomaly (e.g., septate uterus, bicornuate uterus, unicornous uterus) or an acquired intrauterine pathology (e.g., adenomyosis, submucosal leiomyomas, intrauterine adhesions); and (6) Couples with severe male factor infertility (e.g., aspermia, azoospermia, cryptozoospermia).

### Recruitment

Potential participants will be screened from the regular infertile outpatients during their first visit at the reproductive medical center. Eligible patients complying with all inclusion criteria will be invited to receive a free consultation with the doctors or clinical research assistants to discuss the study procedure and all possible outcomes of the trial. Patients who agree to enroll must sign a written informed consent form before any procedures can be carried out. The participants have the right to withdraw their consent and quit the trial at any time for any reason, without any consequences.

### Randomization and masking

Randomization and allocation will be controlled centrally by a web-based niEVT administration database. The randomization will be stratified by fertilization protocol (IVF, ICSI) and by age (< 35, ≥ 35 years). Eligible patients will be independently allocated to either the intervention group (niEVT) or control group (morphological assessment) in a 1:1 ratio using a dynamical stratified blocked randomization algorithm with a block of 6. Regarding patient allocations, the embryologist, research assistants, clinicians, and patients will be blinded.

### Interventions

Oocyte retrieval and fertilization (either IVF or ICSI) will be performed according to the standard protocols of each reproductive medical center. For every eligible patient, all embryos will be cocultured in the same medium drop for the first three days after fertilization (Day 0). On Day 4, embryos will be separately washed 3 ~ 5 times to maximally remove potential maternal/paternal DNA contamination derived from polar cells, cumulus cells, and sperm and then cultured individually to Days 5/6. Morphological grading will be performed on Day 5/6 to determine whether a good-quality (superior to grade 4CB) blastocyst has developed according to Gardner’s criteria [19]. After that, the embryos will be vitrified, and the spent blastocyst culture medium will be collected.

For patients in the intervention (niEVT) group, the embryo transfer order will be determined based on the niPGT-A results (primary standard) and morphological grading results (secondary standard). The embryos will be ranked into three groups: Class 1, with a chromosome status of euploid; Class 2, with an undetermined chromosome status (failed genetic testing or noninformative results); and Class 3, with a chromosome status of aneuploid. Embryos of the same group will be further ranked according to their morphological grades (5AA > 5AB > 5BA > 4AA > 4AB > 4BA > 6AA > 6AB > 6BA > 5BB > 4BB > 6BB > 5AC > 5BC > 4AC > 4BC > 6AC > 6BC > 5CA > 5CB > 4CA > 4CB). Embryos in the same group and with the same morphological grade will be randomly selected for transfer. Based on the rules described above, the embryo transfer order can be achieved. A clinical report with the patient information, embryo IDs, and embryo transfer order will be generated and sent to the clinician. Neither the niPGT-A results nor the morphological grading results will be shown on the report.

For patients in the control (conventional IVF) group, the embryo transfer order will be determined based only on the morphological grading results. A similar clinical report in the same format will be generated and sent to the clinician. The morphological grading results will not be shown on the report.

For patients in both groups, the clinicians will perform a single frozen-thawed blastocyst transfer [20] up to three times according to the embryo transfer order of the clinical report. If the prior embryo transfer fails (no conception for any reason), a second/third embryo transfer will be performed.

### Outcomes

#### Primary outcome

The primary outcome will be the live birth rate after the first embryo transfer. Live birth is defined as the birth of a breathing live-born infant with a heartbeat after 28 completed weeks of gestation.

#### Secondary outcomes

Secondary outcomes, including the clinical pregnancy rate, ongoing pregnancy rate, early miscarriage rate, late miscarriage rate, cumulative live birth rate, ectopic pregnancy rate, and time to pregnancy, are listed in Table 1.

**Table 1:** Primary and secondary outcome measures of this RCT

Outcome	Measure	Definition
Primary outcome	Live birth	Birth of a breathing live-born infant with a heartbeat after 28 completed weeks of gestation
Secondary outcomes	Clinical pregnancy	Presence of intrauterine gestation sacs at 30–35 days after embryo transfer
	Ongoing pregnancy	Presence of a fetal pole with pulsation at 8–10 weeks of gestation
	Early miscarriage	Miscarriage within 12 weeks of embryo transfer
	Late miscarriage	Miscarriage between 12 and 28 weeks of embryo transfer
	Cumulative live birth rate	Birth of a live-born infant conceived within three embryo transfers. Each time a single embryo is transferred is considered.
	Ectopic pregnancy	Pregnancy outside the uterine cavity
	Time to pregnancy	Time interval between the oocyte retrieval date and clinical pregnancy

### Sample size calculation

The sample size was calculated using an online tool (Sample Size Calculator, https://clincalc.com/stats/ samplesize.aspx) based on the following estimation: an estimated 50% and 60% of patients in the control and intervention groups will achieve live births, respectively. The sample size required is 1036 patients (two-sided alpha: 5%, power: 90% [beta: 10%]). Considering an estimated loss to follow-up rate of 10%, the final sample size will be 1152 patients, with each arm comprising 576 patients.

### Adverse events

Training for adverse event (AE) monitoring will be carried out prior to participant enrollment for the members (including the clinicians, research assistants, and nurses) who will perform the study. AE monitoring will be conducted from the start (after providing informed consent) to the end (19 months after oocyte retrieval) of the trial. Adverse events and serious adverse events (SAEs) are defined as any undesirable medical effects (moderate or serious) occurring for participants with any allocation during the study, regardless of whether they are considered to be related to the intervention or not. All SAEs will be reported immediately to the principal investigator and the Trial Management Committee and subsequently documented in detail in the patient’s medical record and niEVT database. Appropriate medical procedures will be taken until the patients recover or are in a stable condition.

### Data collection and management

The clinical data of 1152 patients will be collected, with 576 patients in each group. Data will be collected and stored in two forms, including paper files and electronic files. All clinical data will be first collected and recorded in paper files, including the signed consent form and all types of medical records. Then, the medical records and the niEVT results will be transformed into electronic files and stored in the central niEVT administration database (an online website developed for data management). For privacy protection, patient identification codes will be assigned to every participant and used to link to the clinical data. All paper files will be stored in the reproductive medical centers for five years, and only the local investigators, clinicians, and research assistants will have access to these files. Only the principal investigator and statistician of this trial will have full access to all the data (except for patient privacy information) of the eight reproductive medical centers.

### Statistical analyses

Clinical data from 1152 patients will be collected for statistical analysis, with 576 patients in each group. The statistical analysis will be performed according to the intention-to-treat (ITT) principle [21, 22] using the latest version of R software (https://www.r-project.org/). On the use of two-sided statistical tests with a significance threshold of 5% in this study. The normality of continuous data will first be examined using a graphical method or the Shapiro–Wilk test. Variables with a normal distribution will be presented as the means and standard deviations (SDs). Differences between groups will be analyzed using Student’s *t test*. Skewed variables will be presented as medians and ranges and analyzed using the Mann‒Whitney U test. Categorical and dichotomous data will be presented as numbers (percentages) and analyzed by the chi-square test. For the primary outcome (the live birth rate after the first embryo transfer) and secondary outcome measures (the pregnancy rate, ongoing pregnancy rate, early miscarriage rate, late miscarriage rate, and ectopic pregnancy rate after every embryo transfer; the cumulative live birth rate of the complete IVF cycle; and the time to pregnancy), differences between the intervention and control groups will be investigated using the Cochran‒Mantel‒Haenszel (CMH) test. Multivariable logistic regression will be used for data transformation when necessary.

### Interim analysis

An interim analysis will be performed by the Trial Management Committee after 576 participants have undergone their first embryo transfer and completed three months of follow-up. The results will be reported to the principal investigator for decision-making. The principal investigator will have the right to terminate participant enrollment in the following situations: (1) strong evidence from this study or any other studies demonstrates that the intervention (niEVT) leads to less benefits gained and more AEs; and (2) strong evidence shows that the intervention is superior to the control, or vice versa.

## Discussion

PGT-A was demonstrated to reduce the miscarriage rate and improve the live birth rate after the first embryo transfer in IVF patients, especially for patients of advanced maternal age [23, 24]. niPGT-A is expected to be an alternative to PGT-A [12, 13]. Some clinicians as well as commercial niPGT-A suppliers assume that niPGT-A will bring a similar benefit as PGT-A to patients. However, strong clinical evidence to support or reject this assumption is lacking. The current trial is a well-designed RCT with a large sample size that is expected to fill the knowledge gap.

The study population will be stratified by fertilization method and by age. Based on our preliminary data, embryo washing carefully on Day 4 plus a modified PicoPLEX whole genome amplification protocol (Xu et al., 2022) will help to greatly reduce the genetic testing bias results from maternal/paternal DNA contamination [25]. We speculate that the fertilization method will have limited effects on the final clinical outcomes. However, an inaccurate or even a false niPGT-A result is possible because it is impossible to eliminate the bias completely under the current circumstances. In addition, we are confident that patients of advanced maternal age are very likely to benefit from noninvasive embryo viability testing (niEVT). A higher prevalence of chromosome abnormalities is likely to occur in these patients [26–29]. In addition, a previous study showed that the clinical outcomes of conventional IVF were inferior to those of PGT-A in patients of young maternal age [30]. Based on these results, we speculate that patients of young maternal age will not significantly benefit from the intervention.

Economic evaluation will not be performed in this study. Taking the sampling process into consideration, niPGT-A has an obvious advantage over PGT-A, which relies on special equipment and professional embryologists to perform embryo biopsy [11, 13]. Apart from cost-effectiveness, the most important significance of niPGT-A is that it completely prevents the short-term and long-term risks resulting from embryo biopsy.

To conclude, this large RCT will compare the clinical outcomes between niPGT-A and conventional IVF. This study will answer the question of whether and which patients will benefit from niEVT based on strong clinical evidence.

## Data Availability

The full trial protocol is available upon request to the corresponding author. The trial team is responsible for the final trial dataset. This dataset and the statistical code can be made available upon reasonable request after consideration by the trial management group.

## Declarations

Patients who agree to enroll in this trial must sign a written informed consent form. The participants will have the right to withdraw their consent and quit the trial at any time for any reason, without any consequences.

## Abbreviations

AEs: A training for adverse events
BF: Blastocoel fluid
cfDNA: Cell-free DNA
ChiCTR: Chinese Clinical Trial Registry
CMH: Cochran-Mantel-Haenszel
ET: Embryo transfer
ICSI: Intracytoplasmic sperm injection
ITT: Intention-to-treat
IVF: In vitro fertilization
niEVT: Non-invasive embryo viability testing
niPGT-A: Non-invasive PGT-A
PGT-A: Preimplantation genetic testing for aneuploid
RCT: Randomized controlled trial
SAE: Serious adverse events
SBCM: Spent blastocyst culture medium
SD: Standard deviations
TE-biopsy: Trophectoderm biopsy
TMC: Trial Management Committee
